# Effectiveness of suture anchor and transosseous suture technique in arthroscopic foveal repair of the triangular fibrocartilage complex: a systematic review

**DOI:** 10.1186/s13018-024-04530-4

**Published:** 2024-01-16

**Authors:** Hsuan-Hsiao Ma, Jung-Pan Wang, Chen-Yuan Yang

**Affiliations:** 1 Department of Orthopaedics and Traumatology, Taipei Veterans General Hospital, Taipei, Taiwan; 2 Department of Orthopaedics, School of Medicine, National Yang Ming Chiao Tung University, Taipei, Taiwan; 3 Division of Orthopaedics, Department of Surgery, Taipei Veterans General Hospital Taitung Branch, Taitung, Taiwan; 4 Department of Orthopedic Surgery, Kuang Tien General Hospital, No. 117, Shatian Rd., Shalu Dist., Taichung City, 433 Taiwan; 5 Department of Nursing, Hungkuang University, No. 1018, Sec. 6, Taiwan Blvd., Shalu Dist., Taichung City, 433 Taiwan; 6 Department of Surgery, Taipei Veterans General Hospital Yuli Branch, Hualien, Taiwan

**Keywords:** Foveal repair, Transosseous suture, Suture anchor, TFCC, Triangular fibrocartilage complex, Wrist arthroscopy, Arthroscopic foveal repair

## Abstract

**Background:**

Currently, there were two major surgical methods for arthroscopic triangular fibrocartilage complex (TFCC) foveal repair: suture anchor (SA) and transosseous suture (TOS). The purpose of this systematic review is to examine the relevant outcome improvement and safety of SA and TOS technique.

**Methods:**

Literature review of electronic databases for studies investigating the effects of SA and TOS in patients undergoing arthroscopic TFCC foveal repair was performed. We compared the pre-operative and postoperative functional outcomes, clinical outcomes [pain, range of motion (ROM) and grip strength], and complications of two methods. Minimal clinically important difference (MCID) was used to determine clinically meaningful improvement.

**Results:**

There were 1263 distinct studies identified, with 26 (904 patients) meeting the inclusion criteria. The mean age of participants ranged from 21.4 to 41 years, and the mean follow-up time ranged from 6 to 106 months. Both SA and TOS groups reported significant improvement in the modified mayo wrist score, the disabilities of the arm, shoulder, and hand (DASH) score, quick DASH score, patient-reported wrist evaluation (PRWE) score, and the visual analog scale (VAS) score. According to MCID, all the studies from both groups reporting DASH, quick DASH, PRWE and VAS score achieved clinically meaningful improvement. (MCID: 10 for DASH, 14 for quick DASH, 14 for PRWE and 1.6–18 for VAS). The ROM changes in both groups varied from improvement to deterioration. Grip strength improved in both SA and TOS group. Most complications were self-limited. The reoperation rates in SA and TOS ranged from 0 to 20% and 0 to 27.3%, respectively.

**Conclusions:**

Both SA and TOS technique for arthroscopic TFCC foveal repair could achieve improvement in postoperative functional outcomes, pain, and grip strength with low reoperation rate. However, the ROM improvement was still inconclusive.

**Level of evidence IV:**

Systematic review of level III and IV studies.

**Supplementary Information:**

The online version contains supplementary material available at 10.1186/s13018-024-04530-4.

## Introduction

Triangular fibrocartilage complex (TFCC) injury is the primary cause of ulnar side wrist pain after trauma [1]. Besides pain, these patients usually suffer from range of motion (ROM) impingement, grip strength deterioration and functional impairment. Surgical repair is commonly indicated if symptoms and signs do not improve after conservative treatment with long arm cast or sugar tong splint for 6–8 weeks.

Following Palmar’s work and classification [2, 3], arthroscopic capsular repair for Palmar 1B lesions becomes majority of surgical treatments [4, 5]. With the progress in functional anatomy of TFCC [6], the major stabilizer of distal radio-ulnar joint (DRUJ) is found to be the proximal limb of volar and dorsal DRUJ ligaments [7], not the distal limb responsible for shock absorption. Based on these distal and proximal limb concept, Atezi proposed a treatment-oriented classification for Palmar 1B lesion [8] and emphasized the importance of reattaching the reparable disrupted proximal limb (Atzei class 2/class 3 lesion) back to its foveal insertion to restore DRUJ stability.

The TFCC foveal repair techniques could be divided into two major surgical methods: the suture anchor (SA) technique and transosseous suture (TOS) technique, the former relies on anchor with sutures implanted over fovea and the latter relies on bone tunnel through fovea to pull back the avulsed TFCC proximal component. Because most of the previous studies are retrospective case series with small sample size, the surgical results after each technique remain unclear. Therefore, the purpose of this systematic review is to examine the effectiveness of SA and TOS technique for arthroscopic foveal repair by comparing the pre-operative and postoperative clinical outcomes [pain, grip strength, and range of motion (ROM)], functional outcomes and complications. We hypothesized that both SA and TOS techniques have significant clinical improvement in functional outcomes and similar complications rates.

## Methods

**Fig. 1:**
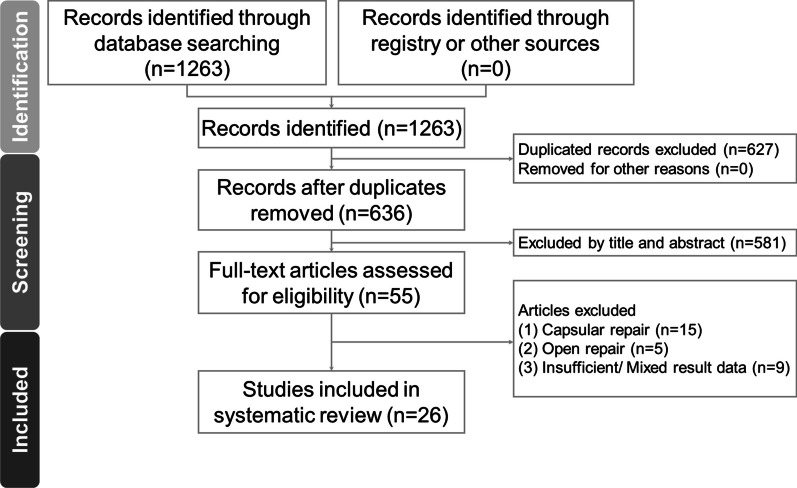
Flow diagram showing the methods to search and identify the included studies

This systematic review adhered to the guidelines of the preferred reporting items for systematic reviews and meta-analyses (PRISMA) [9] (Fig. 1).

### Search strategy

A systematic search of the literature was conducted on 1 June, 2023; we surveyed clinical studies that used arthroscopic fovea repair to treat TFCC injury. PubMed, Embase, Clinical Key, Cochrane CENTRAL, ProQuest, Science Direct, and Web of Science were the primary electronic databases used to find relevant articles. A manual search was also conducted in the reference list of relevant articles and on the clinical trial registry’s website (https://clinicaltrials.gov/). The current systematic review had the following PICO (population, intervention, comparison, and outcome) settings: P, patients undergoing arthroscopic wrist surgery for TFCC injury; I, arthroscopic TFCC fovea repair with suture anchor or transosseous repair; C, preoperative status; O: wrist function, range of motion (ROM), grip strength and pain [Visual Analogue Scale (VAS)]. Two authors searched electronic databases independently using the following keyword combinations: (“arthroscopy” OR “arthroscopic surgery”) AND (“Triangular fibrocartilage complex” OR “TFCC”). During the search, no language restrictions were imposed.

### Inclusion and exclusion criteria

The inclusion criteria were as follows: (1) 18 years of age or older with wrist injury, (2) enrollment in a group undergoing arthroscopic-assisted TFCC foveal repair, and (3) assessment of wrist clinical outcomes before and after surgery. The exclusion criteria were as follows: TFCC open repair, TFCC capsular repair, cadaveric study, child or adolescent study, Atzei class 1 TFCC tear, revision surgery of TFCC repair, mixed results of different arthroscopic repair procedures, and concomitant ulnar shortening osteotomy.

### Data extraction

The abstracts of articles included in our review were screened by two authors. If there was any disagreement about whether the articles were eligible for this systematic review, a decision was reached through the third author opinion. We then obtained the full texts of relevant articles, from which we extracted the relevant data from the tables. The first author’s name, year of publication, study type, patient demographics, clinical and functional outcome measurements, postoperative adverse events, as well as postoperative protocols, including details about immobilization methods and rehabilitation programs, were all extracted.

### Study quality assessment

Included research was evaluated using the methodological index for non-randomized studies (MINORS) [10]. Both of the aforementioned authors worked independently on the process, and any disagreements that arose were resolved through either discussion or a decision made by the third author. The following aspects of the study were assessed: the number of cases and the degree to which they were representative of the population; the selections and definitions of controls; the degree to which cases and controls could be compared; and the ascertainment, consistency, and non-response rates of exposure. In terms of case series, the maximum score is 16; while for comparative studies, the maximum score is 24 referred from MINORS checklist.

If the included article was designed as randomized controlled trial (RCT), the Cochrane Collaboration’s tool [11] was used for study quality assessment.

### Statistical analysis

The primary outcomes were changes in wrist function score before and after arthroscopic-assisted TFCC fovea repair, while secondary outcomes were changes in visual analog scale for pain, ROM, grip strength before and after arthroscopic-assisted TFCC foveal repair. Minimal clinically important difference (MCID) was used to determine clinically meaningful improvement. The average of overall complication, knot irritation, neuropraxia, and reoperation was also presented. The effect size used in this systematic review was the mean differences. Forest plot was used to show the outcome evaluation and also was performed in accordance with the surgical technique (TOS vs. SA). Given as study heterogeneity and the overall studies which are mostly retrospective and non-comparative, the meta-analysis was precluded. As a result, all values were reported as range of mean differences individually. The inter-rater reliability from the degree of the quality assessment was calculated by Cohen’s kappa.

All analyses and graphics were conducted using Comprehensive Meta-analysis Software v4 (Biostat, Englewood, NJ, USA).

## Result

### Literature search

Initially, 1263 relevant articles were identified using the search strategy (Fig. 1). Using the reference management software, Endnote X9 (Clarivate, Cologne, Germany), 627 duplicate records were removed. A total of 581 studies were excluded after reading titles and abstracts, and further 29 studies were excluded after reading the full article: 9 for mixed or insufficient result data, 5 for using open repair and other 15 only doing capsular repair instead of fovea repair. Finally, 26 articles were included in our systematic review. The baseline characteristics of the 26 included studies are summarized in Tables 1 and 2 with SA and TOS technique, respectively. The average age of participants was 31.3 years. The mean age of participants ranged from 21.4 to 41 years and the mean follow up time ranged from 6 to 106 months. The percentage of women was 36%. The studies included 17 case series and 9 comparative studies. Among the 9 comparative studies, seven used a retrospective design, and two used a prospective design [12, 13]. All included studies employed arthroscopic TFCC foveal repair. In addition, the immobilization methods (splint, cast, brace, duration and joint position) and the rehabilitation program (ROM training and strengthening exercises) have been detailed in the supplement (Additional file 1: Table S1 and Additional file 2: Table S2).

**Table 1:** Study characteristics of arthroscopic suture anchor repair of the triangular fibrocartilage complex foveal tear

References	Study design	Case number	Gender (F/M)	Age (years)	From injury to surgery (months)	Follow-up (months)	Outcome measurement				
							VAS	Function score	ROM	Grip	Complication (%)
Kim et al. [34]	Case series	15	4:11	30.5	13.3	29		MMWS, DASH		V	1 (6.7%)
Luchetti et al. [24]	Case control	25	12:13	33	13	31	V	MMWS, DASH, PRWE	PS, FE	V	1 (4.0%)
Atzei et al. [35]	Case series	48	20:28	34	11	33	V	MMWS, DASH	PS, FE	V	5 (10.4%)
Auzias et al. [25]	Case series	24	13:11	41	NR	44	V	Quick DASH, PRWE	PS, FE	V	8 (33.3%)
Kermarrec et al. [41]	Case series	5	2:3	30.8	7.4	29.4	V	Quick DASH, PRWE			0
Hung et al. [15]	Case control	22	14:8	31.5	NR	6	V		FE	V	0
Lu et al. [26]	Case series	16	6:10	40.2	6.4	14.5	V		PS, FE	V	1 (6.3%)
Afifi et al. [12]	RCT	30	10:20	31.8	5.2	24	V	MMWS, Quick DASH, PRWE		V	3 (10.0%)
Yeh et al. [23]	Case series	201	45:156	26.7	2.1	32.6		MMWS, DASH	PS, FE	V	15 (7.5%)

*F* female, *M* male, *VAS* visual analogue scale, *ROM* range of motion, *PS* pronation–supination, *FE* flexion–extension, *NR* not recorded, *RCT* randomized controlled trial, *MMWS* modified Mayo wrist score, *DASH* disabilities of arm, shoulder and hand, *PRWE* patient-rated wrist evaluation

**Table 2:** Study characteristics of arthroscopic transosseous repair of the triangular fibrocartilage complex foveal tear

References	Study design	Case number	Gender (F/M)	Age (years)	From injury to surgery (months)	Follow-up (months)	Outcome measurement				
							VAS	Function score	ROM	Grip	Complication (%)
Iwasaki et al. [36]	Case series	12	6:6	31	8	30	V	DASH	PS, FE	V	2 (16.7%)
Shinohara et al. [27]	Case series	11	4:7	27	9.7	30		MMWS	PS, FE	V	3 (27.3%)
Jegal et al. [37]	Case series	19	8:11	37	6	31		DASH, PRWE		V	9 (47.4%)
Abe et al. [38]	Case control	21	NA	34	8.5	34.4	V	PS, FE		V	0
Park et al. [28]	Case series	16	4:12	29.8	11	31.1	V	MMWS, Quick DASH		V	0
Park and Park [29]	Case series	10	4:6	33.4	8.5	23.5	V	MMWS, Quick DASH		V	0
Dunn et al. [30]	Case series	15	2:13	21.4	3.8	45.6					0
Jung et al. [31]	Case control	42	13:29	35.3	12.4	26.2	V	MMWS, DASH, PRWE	PS, FE	V	0
Park et al. [39]	Case control	80	24:56	27.8	10.5	24	V	MMWS, Quick DASH		V	6 (7.5%)
Hung et al. [15]	Case control	8	4:4	28.4	NA	6	V	FE		V	0
Liu et al. [43]	Case control	25	8:17	28	8	31	V	MMWS, DASH, PRWE	PS, FE	V	1 (4.0%)
Thalhammer et al. [42]	Case series	30	21:9	25	7	106	V	MMWS, DASH			5 (16.7%)
Afifi et al. [12]	RCT	30	15:15	30.2	5.6	24	V	MMWS, Quick DASH, PRWE		V	6 (20.0%)
Gvozdenovic and Simonsen [33]	Case series	44	20:24	32	23	31	V	Quick DASH	PS, FE	V	0
Jung et al. [32]	Case control	40	12:28	34.9	7.6	25	V	MMWS, DASH, PRWE		V	0
Park et al. [40]	Case series	17	5:12	40	8	28.6	V	MMWS, DASH, PRWE	PS, FE	V	0
Yang and Chen [19]	Case series	12	5:7	32	5	53		MMWS, DASH			0
Nam et al. [45]	Case control	66	11:55	24.7	14.4	26.9	V	MMWS, Quick DASH		V	0
Shinohara et al. [44]	Case series	20	11:9	36	NA	17		MMWS	PS, FE	V	2 (10.0%)

*F* female, *M* male, *VAS* visual analogue scale, *ROM* range of motion, *PS* pronation–supination, *FE* flexion–extension, *NR* not recorded, *RCT* randomized controlled trial, *MMWS* modified Mayo wrist score, *DASH* disabilities of arm, shoulder and hand, *PRWE* patient-rated wrist evaluation

### Quality assessment

Methodologic quality assessment of the enrolled studies except Afifi et al. based on MINOR score is presented in Table 3. The mean MINOR score of the non-comparative studies was 9.5. The mean MINOR score of the comparative studies was 17.1. The kappa ratio was 0.79 which was located at the interval of substantial agreement.

**Table 3:** Study characteristics and quality assessment*

References	Level of evidence	Study design	MINORS score
Nam et al. [45]	III	Retrospective comparative study	19
Shinohara et al. [44]	III	Retrospective case series	11
Gvozdenovic and Simonsen [33]	IV	Retrospective case series	11
Jung et al.[32]	III	Retrospective comparative study	18
Park et al. [40]	IV	Retrospective case series	13
Yang and Chen [19]	IV	Retrospective case series	10
Yeh et al.[23]	IV	Retrospective case series	10
Hung et al. [15]	III	Retrospective comparative study	15
Liu et al. [43]	III	Retrospective comparative study	16
Lu et al.[26]	IV	Retrospective case series	8
Thalhammer et al. [42]	IV	Retrospective case series	12
Auzias et al.[25]	IV	Retrospective case series	8
Kermarrec et al. [41]	IV	Retrospective case series	8
Park et al. [39]	III	Retrospective comparative study	19
Dunn et al.[30]	IV	Retrospective case series	8
Jung et al.[31]	III	Retrospective comparative study	18
Abe et al. [38]	III	Retrospective comparative study	16
Park et al. [28]	IV	Retrospective case series	9
Park and Park [29]	IV	Retrospective case series	9
Jegal et al. [37]	IV	Retrospective case series	8
Atzei et al. [35]	IV	Retrospective case series	8
Luchetti et al.[24]	III	Prospective comparative study	16
Kim et al. [34]	IV	Retrospective case series	11
Shinohara et al. [27]	IV	Retrospective case series	10
Iwasaki et al. [36]	IV	Retrospective case series	9

*MINORS* methodological index for non-randomized studies

Afifi et al. [12], which was designed as RCT, was assessed by Cochrane Collaboration’s tool. All the domain was showed low risk of bias.

### Wrist function between preoperative and postoperative status

**Fig. 2:**
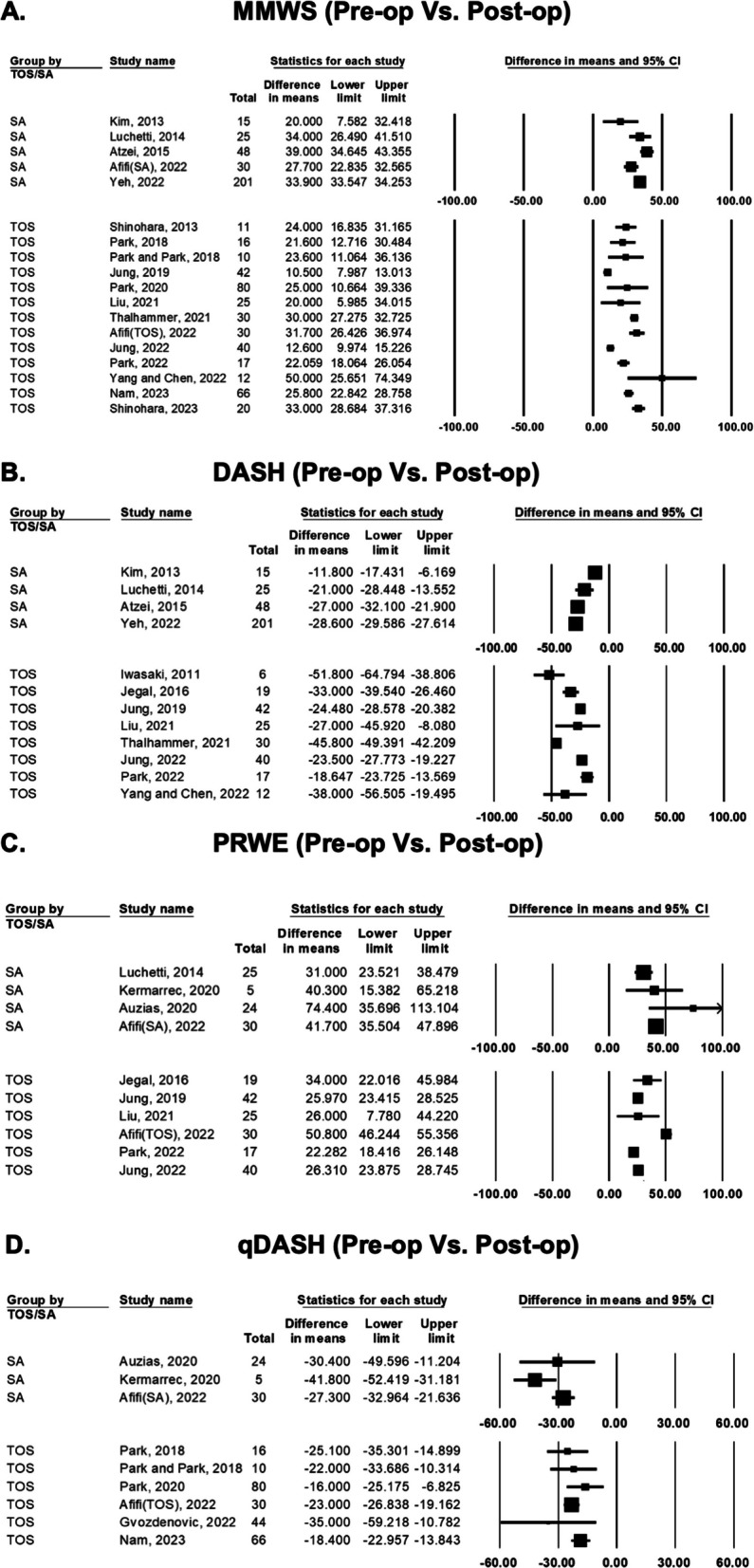
Forest plot comparing preoperative and postoperative function score of transosseous (TOS) group and suture anchor (SA) group: Modified Mayo Wrist Scores (MMWS) (**A**); The disability of the arm, shoulder and hand (DASH) score (**B**); patient-rated wrist evaluation (PRWE) (**C**); and quick DASH (q-DASH) score (**D**)

There was total 22 studies reporting the wrist function scores before and after surgery (Fig. 2). For modified mayo wrist score (MMWS), the difference between preoperative and postoperative status were compared in 17 studies. The range of difference in means of SA group was 20.0–39.0. Among these 5 SA studies, all reported significant improvement. The range of difference in means of TOS group was 10.5–50.0. Among these 13 TOS studies, all reported significant improvement.

For DASH score, the difference between preoperative and postoperative status was compared in 12 studies. The range of difference in means of SA group was − 28.6 to − 11.8. Among these 4 SA studies, all reported significant improvement and reached minimal clinically important difference (MCID:10 for DASH)^13^. The range of difference in means of TOS group was − 51.8 to − 18.6. Among these 8 TOS studies, all reported significant improvement and reached minimal clinically important difference.

For PRWE, the difference between preoperative and postoperative status were compared in 9 studies. The range of difference in means of SA group was 31.0–74.4. Among these 4 SA studies, all reported significant improvement and reached minimal clinically important difference (MCID:14 for PRWE)^13^. The range of difference in means of TOS group was 22.2–50.8. Among these 6 TOS studies, all reported significant improvement and reached minimal clinically important difference.

For quick DASH score, the difference between preoperative and postoperative status were compared in 8 studies. The range of difference in means of SA group was − 41.8 to − 27.3. Among these 3 SA studies, all reported significant improvement and reached minimal clinically important difference (MCID:14 for quick DASH score)^13^. The range of difference in means of TOS group was − 35.0 to − 16.0. Among these 6 TOS studies, all reported significant improvement and reached minimal clinically important difference.

### Difference in VAS for pain between preoperative and postoperative status

**Fig. 3:**
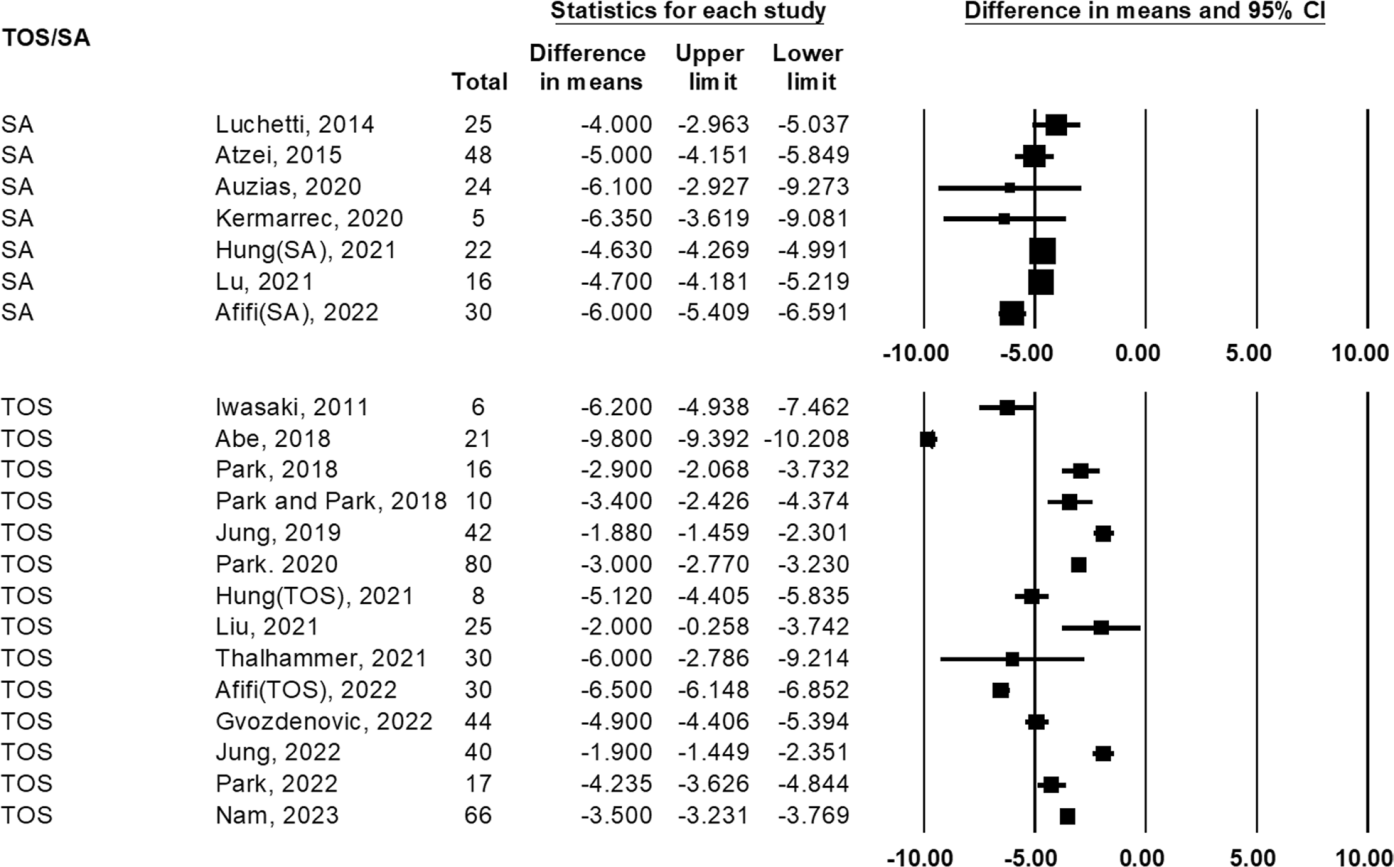
Forest plot comparing preoperative and postoperative visual analog scale (VAS) of transosseous (TOS) group and suture anchor (SA) group

For the VAS score, the difference between preoperative and postoperative status was compared in 19 studies (Fig. 3). The range of difference in means of SA group was − 6.35 to − 4.00. Among these 7 SA studies, all reported significant improvement. All the studies reached minimal clinically important difference of VAS (MCID, 1.6–1.8) and reached substantial clinical benefit (SCB, 2.2–2.6) [14]. The range of difference in means of TOS group was − 9.80 to − 1.88. Among these 14 TOS studies, all reported significant improvement. All the studies reached minimal clinically important difference of VAS (MCID, 1.6–1.8) and 11 of 14 studies reached substantial clinical benefit (SCB, 2.2–2.6).

### Difference in range of motion (ROM) between preoperative and postoperative status

**Fig. 4:**
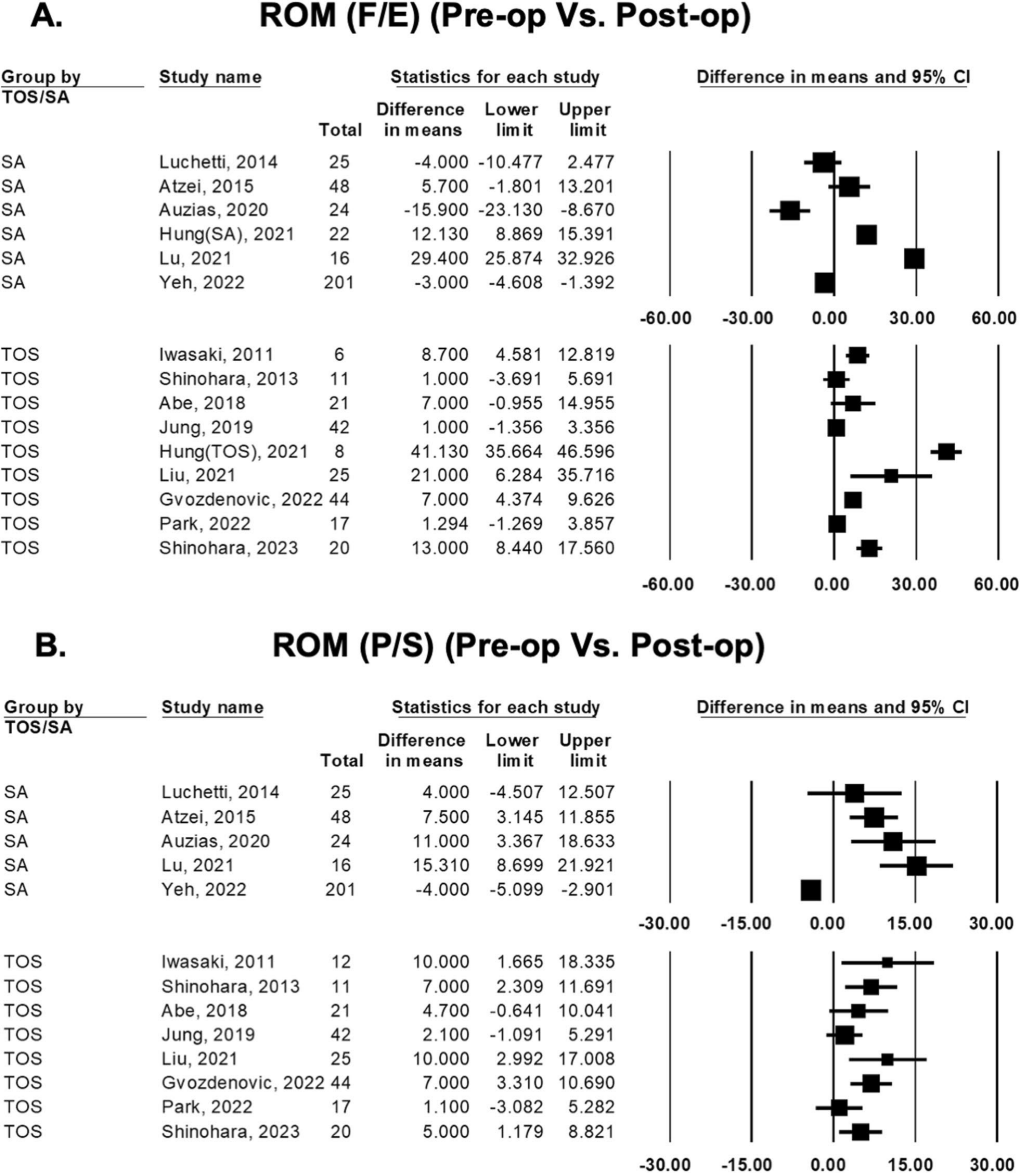
Forest plot comparing preoperative and postoperative range of motion (ROM) of transosseous (TOS) group and suture anchor (SA) group: flexion/extension (F/E) (**A**); and pronation/supination (P/S) (**B**)

The flexion–extension ROM change between preoperative and postoperative status was compared in 14 studies (Fig. 4). The range of difference in means of SA group was − 15.9° to 29.4°. Among these 6 SA studies, 2 reported significant improvement, 2 reported no significant improvement and 2 reported significant deterioration. The range of difference in means of TOS group was 1.0° to 41.1°. Among these 9 TOS studies, 5 reported significant improvement, and 4 reported no significant improvement.

The pronation–supination ROM change between preoperative and postoperative status were compared in 13 studies. The range of difference in means of SA group was − 4.0° to 15.3°. Among these 5 SA studies, 3 reported significant improvement, 1 reported no significant improvement and 1 reported significant deterioration. The range of difference in means of TOS group was 1.10°–10.00°. Among these 8 TOS studies, 5 reported significant improvement, and 3 reported no significant improvement.

### Difference in grip strength between preoperative and postoperative status

**Fig. 5:**
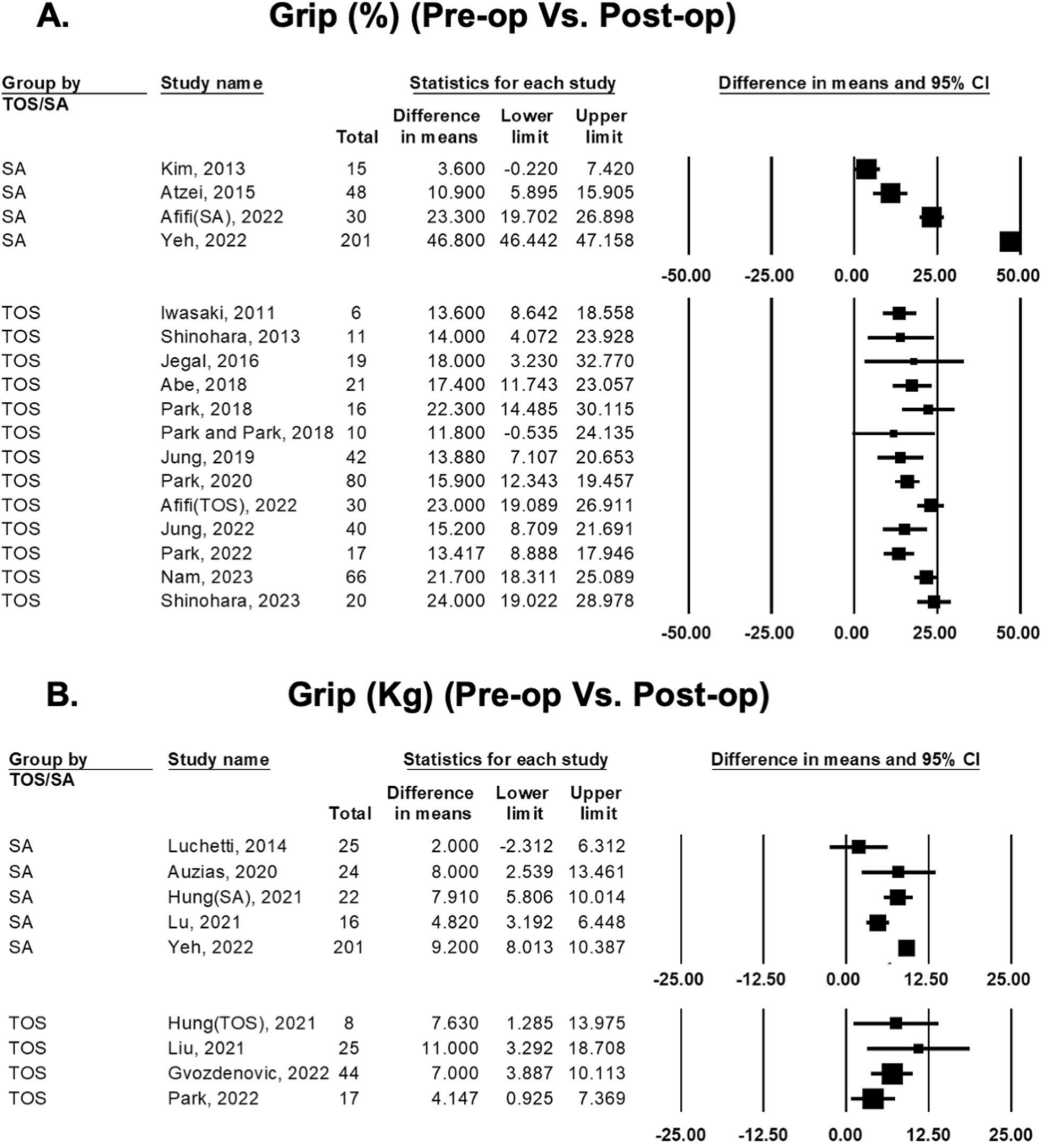
Forest plot comparing preoperative and postoperative grip strength of transosseous (TOS) group and suture anchor (SA) group: percentages of contralateral wrist (**A**); and kilogram data of the operated wrist (**B**)

For grip strength presented as percentages of contralateral wrist, the difference between preoperative and postoperative status were compared in 16 studies (Fig. 5). The range of mean differences of SA group was from 3.6 to 46.8%. Among these 4 SA studies, 3 reported significant improvement, 1 reported no significant improvement. The range of mean differences of TOS group was from 11.8 to 24.0%. Among these 13 TOS studies, 12 reported significant improvement, 1 reported no significant improvement.

For grip strength presented as kilogram data of the operated wrist, the difference between preoperative and postoperative status were compared in 8 studies. The range of mean differences of SA group was from 2.0 to 9.2 kg. Among these 5 SA studies, 4 reported significant improvement, 1 reported no significant improvement. The range of mean differences of TOS group was from 4.1 to 11.0 kg. Among these 4 TOS studies, all reported significant improvement.

### Complication and reoperation

**Fig. 6:**
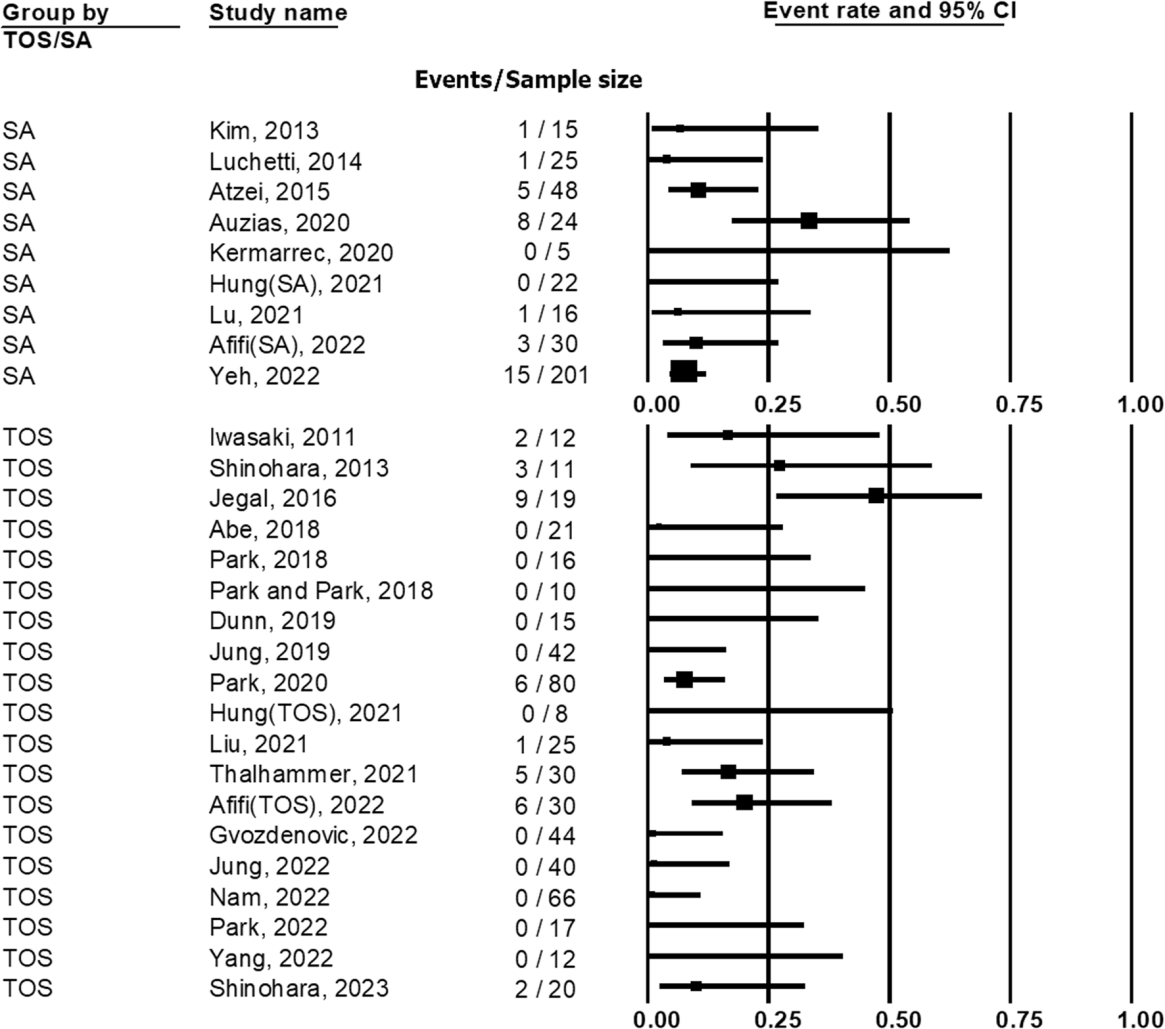
Forest plot demonstrating the complication rate of transosseous (TOS) group and suture anchor (SA) group

**Fig. 7:**
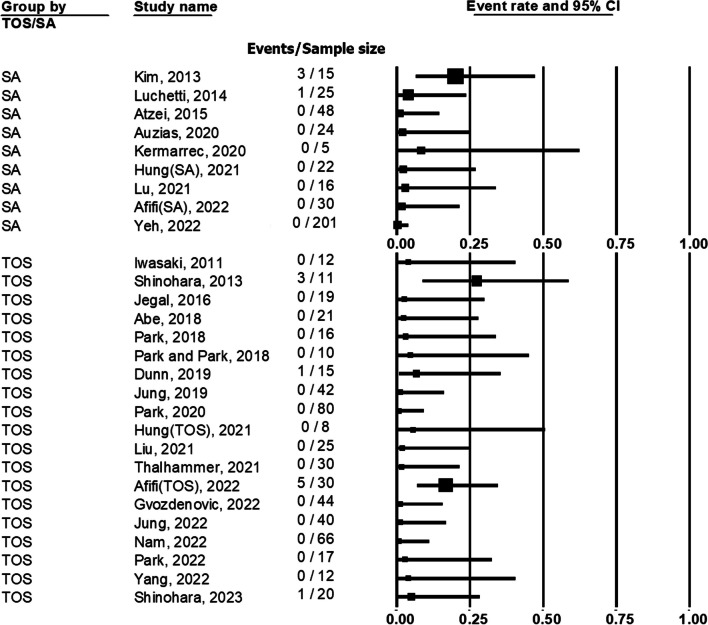
Forest plot demonstrating the reoperation rate of transosseous (TOS) group and suture anchor (SA) group

The complications and reoperation events of SA group were recorded in 9 studies. There were 2 studies revealing no complication after the surgery in SA group. The complication rate in SA group ranged from 0 to 33.3% (Fig. 6). Neuropraxia of dorsal cutaneous branch of ulnar nerve ranged from 0 to 33.3% and suture knots irritation ranged from 0 to 6.7%. The reoperation rate ranged from 0 to 20% (Fig. 7).

The complications and reoperation events of TOS group were recorded in 19 studies. There were 11 studies stated there was no complication after the surgery. The complication rate in TOS group ranged from 0 to 47.4% (Fig. 6). Neuropraxia of dorsal cutaneous branch of ulnar nerve ranged from 0 to 10% and suture knots irritation ranged from 0 to 47.4%. The reoperation rate ranged from 0 to 27.3% (Fig. 7).

## Discussion

In this systematic review, we compare the preoperative and postoperative clinical and functional outcome variables of the arthroscopic TFCC foveal repair studies, and the results proved the hypothesis and showed significant improvement of the postoperative functional score, pain, and grip strength in both SA group and TOS group.

In the literature review, there were only two case control studies comparing the effectiveness of SA and TOS for arthroscopic TFCC foveal repair, Hung et al. presented first retrospective study [15] and Afifi et al. presented first prospective randomized controlled trial of 2 equal groups and all surgeries performed by same surgeon [12]. Both studies showed comparable outcomes in pain relief, and grip strength improvement. In this systematic review, the clinical results, functional outcomes and complications of the SA and TOS technique for arthroscopic foveal repair of TFCC were comprehensively evaluated.

The Forest plot in our study showed improved postoperative function scores, VAS and grip strength after surgical repair of TFCC foveal rupture with suture anchor or TOS technique. This result is consistent with previous case control studies comparing these two techniques [12, 15] or systematic review focusing on arthroscopic transosseous foveal repair [16]. Reattachment of avulsed proximal limb of TFCC to its foveal insertion could effectively restore DRUJ stability, thus reduce the pain, grip strength weakness and functional impairment caused by unstable DRUJ. The concept of foveal repair for Atzei class 2 complete tear or Atzei class 3 proximal tears [8] explained the inconsistent surgical results after capsular repair for Palmar 1B tear lesions with DRUJ instabilities [17]. Arthroscopic transosseous suture method was first introduced by Iwasaki in 2009 with single suture strand [18] and further modified with more comprehensive suture configuration [19] as recent cadaveric studies demonstrated the three-dimensional morphology of the TFCC foveal insertion [20, 21]. Arthroscopic suture anchor repair utilizes one to two non-absorbable sutures to reattach the disrupted TFCC proximal limb to the anchoring fovea insertion site. Both techniques were reliable according to our study results.

Restoring the DRUJ stability is an important outcome parameter after TFCC foveal repair surgeries, and clinically Ballottement test was used to examine the DRUJ stability by checking the volar-dorsal translation of ulnar head while firmly holding the distal radius and carpal bones in position. A biomechanical study has shown [22] TOS technique showed greater resistance to ulnar translation than suture anchor technique in cadaveric model of TFCC foveal tears. We tried to involve DRUJ stability as one of the outcome variables, but found it not possible to be compared in the systematic review because result of Ballottement test was difficult to be presented as the percentage or distance of ulnar translation for data analysis.

As for the ROM comparison between preoperative and postoperative status, both the flexion/extension arc and pronation/supination arc change in the suture anchor group and TOS group were inconclusive. Postoperative protocols, encompassing immobilization methods, duration, positioning, as well as range of motion (ROM) exercises and strengthening exercises, play a pivotal role in facilitating the patient's recovery. However, the rehabilitation protocols differed among the studies included in both groups. The decreased ROM might result from prolonged immobilization and delayed wrist rehabilitation for 4–8 weeks after surgery in postoperative protocol of Yeh [23], Luchetti [24] and Auzias [25]. On the contrary, Lu [26] of the suture anchor group and most of TOS group studies starts wrist flex/extension training 2–4 weeks after surgery to reduce immobilization related scarring and stiffness. Prospective randomized controlled trial is needed to clarify the exact relationship between surgery, immobilization protocol, and ROM improvement.

The overall complication rate in SA group was higher than TOS group (8.8% vs. 6.6%), which would appear to differ from previous comparative studies [12, 15]. In complications of SA group, most cases (15/34 = 44.1%) result from neuropraxia injury of ulnar nerve dorsal cutaneous branch, which were almost self-limited in 2–4 months. The cause of cutaneous nerve injury might result from extreme supination position required to insert the suture anchor into correct fovea insertion site through direct fovea portal, which incision was usually not large enough to prevent over-traction of the surrounding cutaneous nerve. Neuropraxia occurred much less In TOS group might because the operated wrist was almost kept in neutral of slight supination position during whole procedure, and the medial longitudinal incision for bone tunnel preparation and sutures retrieval provided more space for surgeon to identify and protect the dorsal cutaneous branch of ulnar nerve.

In complications of TOS group, most cases (17/34 = 50.0%) result from suture knots irritation, which need surgical removal in total 8 cases [12, 27]. This is because the suture knots were usually tied around ulnar cortex of bony tunnel entrance underneath a thin layer of soft tissue and skin. To reduce the knots irritation, proper repairing the retinaculum [19] or buried the sutures with knotless suture anchor [28–33] should be considered. Contrarily, the SA group have much fewer complications of knot irritation because the knots were tied over TFCC and hardly be felt outside the radiocarpal joint. Although there is difference in occurrence rates and major cause of complication, the reoperation rates were similarly low in both groups (SA 1.0% vs. TOS 1.9%).

### Limitation

The limitation of this systematic review was that most of the included studies were case series, lack of high-quality case control studies or prospective randomized controlled trials. Furthermore, there are no universal forms of function scores evaluation (MMWS, DAHS, quick DASH, PRWE), but there are at least three studies included in each subgroup analysis of functional scores. DRUJ stability was not included for outcome analysis due to no objective data for ulnar translation of Ballottement test. Otherwise, the details in each surgical technique group (ex. transosseous tunnel number, tunnel size, suture number, absorbable or non-absorbable materials, suture configuration and postoperative protocols) could not be standardized and might cause bias in analysis. While comparing intraoperative data, such as surgery time and costs, could yield meaningful insights, these results were not presented due to a lack of relevant data from the enrolled studies. We anticipate conducting further investigations when updated data becomes available.

## Conclusions

Both SA and TOS techniques for arthroscopic TFCC foveal repair could achieve improvement in postoperative functional outcomes, pain, and grip strength with low reoperation rate. However, the ROM improvement was still inconclusive.

More prospective randomized controlled trials are needed to further clarify the effectiveness and safety of SA and TOS techniques in arthroscopic foveal repair of the triangular fibrocartilage complex.

### Supplementary Information

**Additional file 1: Table S1.** Postoperative protocol of arthroscopic suture anchor repair of the triangular fibrocartilage complex foveal tear.

**Additional file 2: Table S2.** Postoperative protocol of arthroscopic transosseous repair of the triangular fibrocartilage complex foveal tear.

## Data Availability

Data availability is not applicable to this article as no new data were generated or analyzed in this study.
