# Supplementary material for: Effectiveness of suture anchor and transosseous suture technique in arthroscopic foveal repair of the triangular fibrocartilage complex: a systematic review
Source: J Orthop Surg Res. 2024 Jan 16;19:72. doi: 10.1186/s13018-024-04530-4 (PMC10790567; doi:10.1186/s13018-024-04530-4)
Supplement: Supplementary file 1 — Additional file 1: Table S1. Postoperative protocol of arthroscopic suture anchor repair of the triangular fibrocartilage complex foveal tear. [file 13018_2024_4530_MOESM1_ESM.docx]

Supplementary Table S1. Postoperative protocol of arthroscopic suture anchor repair of the triangular fibrocartilage complex foveal tear

| Author, year | Immobilization | Rehabilitation |
| --- | --- | --- |
| Kim et al, 2013 | Long arm splint for 4 weeks (45°supination), followed by a short arm brace for an additional 2 weeks | At 6 weeks postoperatively, wrist motion was allowed with rehabilitation. |
| Luchetti et al, 2014 | -Above-elbow cast for 1 week (elbow in 90° of flexion, slight supination (45°) and the wrist in a neutral position)  -Thermoplastic long arm splint for 30 days maintaining the same position  -Wrist splint for an additional 4 weeks | -After 7 days, flexion and extension elbow exercises were started under the supervision of the hand therapist with guided prevention of any forearm pronation or supination movements.  -After 30 days postoperatively, an active and passive hand therapy program was started allowing prono-supination without resistance.  - From the ninth to twelfth post-operative weeks, muscle strengthening exercises against resistance were added to the wrist rehabilitation program to reestablish functional wrist stability, proprioception and improve the range of motion of the wrist. Sporting activities were allowed only after 6 months and after completion of a wrist rehabilitation program. |
| Atzei et al, 2015 | Long-arm cast for 1 week (neutral rotation or slight (45°) supination), followed by a Münster-type thermoplastic splint for 3 weeks, which restricts forearm rotation and allows limited elbow flexion/extension | -Proprioceptive rehabilitation with isometric exercises, co-contraction of the pronator quadratus and extensor carpi ulnaris and flexion/extension of the wrist starts at 4weeks.  -Six weeks postoperatively, progressive forearm rotation is allowed and the patient can remove the splint during the  day. Full ROM and resisted wrist and hand strengthening exercises are not permitted until the patient recovers consistent proprioceptive control.  -Daily activities can be resumed as soon as 2 months postoperatively. However, sport and heavy work tasks usually require more than 3 months, according to the recovery of forearm muscle strength and endurance. |
| Auzias et al, 2020 | Short-arm volar splint for 3 days, followed by a Sarmiento splint for 6 weeks (prevent pronation-supination but allow elbow flexion-extension) | Rehabilitation started at the 6th week postoperative. |
| Kermarrec et al, 2020 | Brachioantebrachial splint for 3 weeks (neutral position), followed by a removable wrist splint for 3 more weeks | -Digital range-of-motion exercises are initiated immediately. ROM and strengthening exercises of the forearm and wrist are initiated at 6weeks.  -Manual activities and sports can be resumed at 2 months. |
| Hung et al, 2021 | A palmar reinforcement brace keeping forearm in neutral position | N/A |
| Lu et al, 2021 | -Long arm splint for 1 week (elbow flexion 90° and forearm neutral position), followed by removable brace for 6 weeks | -After 3 weeks, allow flexion-extension, ulnar-deviation, forearm rotation; 3 times a day and each time 10 minutes  -After 3 months, start strengthening exercise  -Within 6 months, avoid strenuous activities |
| Afifi et al, 2022 | Above-elbow cast for 3 weeks (elbow at 90° and the forearm in neutral rotation), followed by Müenster-type cast for further 3 weeks (limit the forearm rotation while allowing some elbow flexion/extension) | -Early digital motion was encouraged.  -Six weeks after surgery, the cast was removed, and unrestricted motions were allowed with progressive strengthening exercises. |
| Yeh et al, 2022 | Wrist brace for 6 weeks (fix the wrist in a neutral position) | -The subsequent occupational hand therapy program involved active and passive range of motion  and isometric exercises.  -After 1 month of occupational therapy, the patients progressed to therapy that incorporated strengthening and work simulation exercises. |

N/A: not available in the article; ROM: range of motion
