# Supplementary material for: Effectiveness of suture anchor and transosseous suture technique in arthroscopic foveal repair of the triangular fibrocartilage complex: a systematic review
Source: J Orthop Surg Res. 2024 Jan 16;19:72. doi: 10.1186/s13018-024-04530-4 (PMC10790567; doi:10.1186/s13018-024-04530-4)
Supplement: Supplementary file 2 — Additional file 2: Table S2. Postoperative protocol of arthroscopic transosseous repair of the triangular fibrocartilage complex foveal tear. [file 13018_2024_4530_MOESM2_ESM.docx]

Supplementary Table S2. Postoperative protocol of arthroscopic transosseous repair of the triangular fibrocartilage complex foveal tear

| Author, year | Immobilization | Rehabilitation |
| --- | --- | --- |
| Iwasaki et al, 2011 | Long arm cast for 4 weeks (45° of supination, prevent rotation), followed by a removable wrist brace for an additional 2 weeks | -At 6 weeks postoperatively, vigorous rehabilitation of the wrist and forearm was started. |
| Shinohara et al, 2013 | Long arm cast for 3 to 4 weeks (elbow flexion 90°, forearm in neutral rotation, and wrist in neutral), followed by wrist brace for 8 weeks | -After removal of cast, active ROM exercises for the wrist and forearm were started. |
| Jegal et al, 2016 | Long arm splint/cast for total 4 weeks (elbow flexion 90°, forearm in neutral rotation or semisupinated position), followed by a removable long arm brace until full motion recovery | -After removal of the splint or cast, patients were encouraged to begin active assisted range-of-motion exercises of the wrist.  -Strengthening exercises of the forearm and wrist are started at 12 weeks and heavy physical activities are allowed at 6 months after surgery. |
| Abe et al, 2018 | Long arm cast for 2 weeks (elbow flexion 90°, forearm in neutral rotation), followed by a short arm cast for an additional 2 weeks | -Gentle ROM exercise including rotation of the forearm was started at 4 weeks after surgery.  -Grip strengthening was started at 2 months. The patients were instructed that they could return to preoperative sports or work 3 to 6 months after surgery |
| Park et al, 2018 | Long arm cast for 6 weeks (neutral to 30° forearm supination), followed by a removable rigid wrist brace for an additional 2 weeks | -After the removal of the cast, active ROM exercises were initiated. The aim of physical therapy was to achieve full pronation–supination range at 8–9 weeks after the surgery.  -Isometric strengthening exercises were initiated about 3 months after the surgery. The patients were permitted to resume contact sport activities at 6 months after the surgery. |
| Park and Park, 2018 | Long arm cast for 6 weeks (30° forearm supination), followed by a removable wrist brace for an additional 2 weeks | -After removal of the cast, active ROM exercise was started. The target of physiotherapy was to achieve full pronation supination range of wrist motion at 8–9 weeks after the operation.  -Isometric strengthening exercise was initiated about 3 months after the surgery. The patient was permitted to resume contact or heavy-lifting sports activities 6 months after the surgery. |
| Dunn et al, 2019 | Long arm orthosis for 2 weeks (elbow flexion 90°, forearm in neutral rotation, and wrist in neutral), followed by a Munster cast for an additional 4 weeks | -Active ROM exercises for the wrist and forearm are started after the cast is removed and under the supervision of a certified hand therapist. |
| Jung et al, 2019 | Long-arm cast for 4 weeks (elbow flexion 90°, forearm from neutral to 30° supination), followed by a removable wrist brace for an additional 4 weeks | -Active ROM wrist and forearm exercises were allowed after cast removal.  -Strengthening exercises were started at 8 weeks. |
| Park et al, 2020 | Long-arm orthosis cast for 6 weeks, followed by a  removable short-arm orthosis for 2 weeks | -Exercises for wrist ROM were initiated immediately after removal of the orthosis.  -Patients were permitted to resume contact sport activities 6 months after surgery |
| Hung et al, 2021 | Long arm splint (forearm in neutral position) | N/A |
| Liu et al, 2021 | Sugar tong splint for 3 weeks (semi-supination) | -From week 4, the patient begins gradual active flexion–extension and pronation–supination up to 45°.  -From week 7, the splint could be removed completely, pronosupination range increased and weight loading commenced. |
| Thalhammer et al, 2021 | Sugar tong splint for 6 weeks | -After removing splint, hand therapy was started to gain free motion by active exercises, with partial weightbearing for another 6 weeks. Thereafter, strengthening exercises were allowed and return to manual labor and sports was initiated as desired by the patients. |
| Afifi et al, 2022 | Above-elbow cast for 3 weeks (elbow at 90° and the  forearm in neutral rotation), followed by Müenster-type cast for further 3 weeks (limit the forearm rotation while allowing some elbow flexion/extension) | -Early digital motion was encouraged.  -After removing cast, unrestricted motions were allowed with progressive strengthening exercises. |
| Gvozdenovic and Simonsen, 2022 | Sugar tong splint for 2 weeks, followed by a removable elbow orthosis with limited rotation for further 4 weeks | -Occupational therapy included gradual movement and weight-increasing exercises.  -Full weight-bearing activities were allowed at 3 months.  -Contact sports and heavy, full body weight-bearing activities without orthosis were allowed at 6 months |
| Jung et al, 2022 | Group I: Long arm cast (neutral position)/ Group II: Short arm cast (45° semi-supination) for 4 weeks, followed by a removable high support wrist brace for further 4 weeks | -Active ROM exercises for the wrist and forearm were allowed after the cast was removed. The aim of physiotherapy was to achieve a full ROM at eight to nine weeks after the surgery.  -Strengthening exercises were then started. The patients were permitted to start sporting activities three months after the surgery. |
| Park et al, 2022 | Long arm splint for 4 weeks | -After removing splint, an active and passive hand therapy program was started, allowing supination-pronation without resistance.  -Muscle-strengthening exercises against resistance were added at 3 months after operation.  -Sporting activities were allowed at 6 months after surgery |
| Yang and Chen, 2022 | Sugar tong splint for 3 weeks | - Midrange forearm rotation and unlimited wrist flexion and extension are allowed in the following 5 weeks after discarding the splint while maximal forearm supination and pronation are avoided during the initial 2 months after surgery. A full range of forearm rotation, wrist flexion–extension and normal activity is achieved about 3 months after the surgery.  -Progressive forearm strengthening exercises start 1 month after surgery. Maximal loading is not encouraged within the 6 months after surgery until more robust healing. |
| Nam et al, 2023 | NA | NA |
| Shinohara et al, 2023 | Sugar tong orthosis for 3 weeks, followed by a wrist brace for 3 additional weeks | -Active and passive wrist flexion and extension exercises were initiated after 3 weeks, and pronation and supination exercises were initiated after 6 weeks.  -Strength training and return to sports were permitted 2 months after surgery |

N/A: not available in the article; ROM: range of motion
